# Immune aging in annual killifish

**DOI:** 10.1186/s12979-024-00418-3

**Published:** 2024-03-08

**Authors:** Gabriele Morabito, Alina Ryabova, Dario Riccardo Valenzano

**Affiliations:** 1 Leibniz Institute on Aging, Fritz Lipmann Institute, Jena, Germany; 2 Friedrich Schiller University, Jena, Germany; 3 Cluster of Excellence Balance of the Microverse, Friedrich Schiller University, Jena, Germany

**Keywords:** Aging, Immunity, Killifish, Gerontology, Teleosts

## Abstract

Turquoise killifish (*Nothobranchius furzeri*) evolved a naturally short lifespan of about six months and exhibit aging hallmarks that affect multiple organs. These hallmarks include protein aggregation, telomere shortening, cellular senescence, and systemic inflammation. Turquoise killifish possess the full spectrum of vertebrate-specific innate and adaptive immune system. However, during their recent evolutionary history, they lost subsets of mucosal-specific antibody isoforms that are present in other teleosts. As they age, the immune system of turquoise killifish undergoes dramatic cellular and systemic changes. These changes involve increased inflammation, reduced antibody diversity, an increased prevalence of pathogenic microbes in the intestine, and extensive DNA damage in immune progenitor cell clusters. Collectively, the wide array of age-related changes occurring in turquoise killifish suggest that, despite an evolutionary separation spanning hundreds of millions of years, teleosts and mammals share common features of immune system aging. Hence, the spontaneous aging observed in the killifish immune system offers an excellent opportunity for discovering fundamental and conserved aspects associated with immune system aging across vertebrates. Additionally, the species' naturally short lifespan of only a few months, along with its experimental accessibility, offers a robust platform for testing interventions to improve age-related dysfunctions in the whole organism and potentially inform the development of immune-based therapies for human aging-related diseases.

## Main text

In a visionary novel about the desperate quest for human immortality in 1930s Los Angeles, British novelist Aldous Huxley imagines a group of scientists running a secret experimental program focused on researching the secrets of long life in – among other animals – long-lived carps [1]. Three decades later, Los Angeles-based gerontologist Roy Walford, and UK-based experimental gerontologist Alex Comfort, pioneered the experimental use of teleosts to investigate the physiologic and histologic basis of organismal aging [2, 3]. Alex Comfort’s work refuted the hypothesis that ever-growing teleosts might escape the aging process achieving to live indefinitely [2, 4]. Walford’s work, on the other hand, focused on annual killifishes as a naturally short-lived vertebrate model, a resource for both fundamental insights into vertebrate’s aging patho-physiology [3, 5], as well as an experimental platform to test experimental interventions that extend lifespan [6, 7]. The work initiated by Comfort, Walford and colleagues, started to shed light on the spontaneous onset of aging phenotypes among Cyprinodontiformes, including annual killifishes [8]. Remarkably, these small-sized teleosts appeared to recapitulate several aspects of mammalian aging, including the age-dependent increase in individual mortality, spontaneous onset of cancer, pathological lesions in the liver, as well as thymus involution [9–12]. Killifish aging appeared to be characterized by stark age-dependent changes in the main immune organs’ histology, including the thymus and the kidney marrow – the mammalian bone marrow fish equivalent [11]. Noteworthy, Roy Walford pioneered the “immunologic theory of aging” [13, 14], which postulates that immune system aging contributes to systemic aging. The insights obtained by Walford’s studies about aging in killifish might have contributed to his views on the role of immune system during organismal aging and aging-related pathologies.

### Evolutionary ecology of annual killifish

Annual killifish are adapted to extreme environments characterized by alternating dry and wet seasons throughout the year [15, 16]. Annual killifish inhabit temporary ponds within seasonal rivers' drainage basins, which experience periodic flooding during brief rainy seasons. Throughout dry periods, these fish endure as desiccation-resistant diapausing embryos, capable of surviving and developing within the arid mud. During the brief rainy seasons, killifish hatch and reach sexual maturity in as little as two weeks, which is faster than any other recorded vertebrate [17]. Adult killifish survival is constrained by the limited availability of water during brief rainy seasons, as pond desiccation results in adult fish death. Other causes of extrinsic mortality in annual killifish are predators (largely birds and aquatic insects), as well as parasites [18]. While annual killifish inhabit ephemeral habitats and are ecologically constrained to a brief adult life in nature, they also display a short lifespan when raised in captivity.

To date, it is still not completely clear what are the mechanistic and evolutionary causes underlying the aging phenotypes rapid onset that constrain lifespan in killifish, making them significantly shorter-lived compared to other teleosts, including other closely related Cyprinodontiformes. One hypothesis that would explain the short natural lifespan in annual killifish is that the same biological mechanisms that control embryonic diapause and rapid sexual maturation come with a cost in late life, hence constraining adult survival. However, to date there is no substantial support towards this type of evolutionary trade-off in killifish. An alternative scenario that would explain the onset of aging-related dysfunctions, as well as the short adult lifespan of annual killifish, is the accumulation of genome-wide deleterious gene variants exacerbated by extensive population bottlenecks and small effective population size [19, 20]. However, while extensive genome-wide relaxation of purifying selection correlates with short adult lifespan and rapid aging in annual killifish, small effective population size per se might not be the only factor explaining short killifish lifespan. In fact, longer-lived non-annual killifish, such as *Pachypanchax playfairii*, which also underwent extreme population bottlenecks, live several years, and do not show the same spectrum of aging-related dysfunctions typical of short-lived annual killifish [21]. It is therefore plausible that other ecologically relevant factors, e.g. population fragmentation and an upper limit to yearly water availability, might play an important role in the evolution of short natural lifespan observed in annual killifishes.

### Annual killifish in the genus *Nothobranchius*, a natural model of immune aging

At the end of the 1970s and at the beginning of the 1980s, gerontologists recognized that annual killifish in the genus *Nothobranchius* undergo remarkable spontaneous changes in adult-specific immune features, in parallel with the onset of malignant transformations [9–11]. These initial observations set the basis for follow-up studies aimed at dissecting the biological mechanisms involved in immune system aging in killifish.

Recent work, still published as a preprint, shows that the main hematopoietic organ of turquoise killifish – the kidney marrow – presents typical vertebrate immune cell lineages, distinct in myeloid and lymphoid lineages [22]. Thanks to the possibility to characterize cell type identity through single-cell RNA Sequencing, it has recently become possible to study cellular and transcriptional changes in aged immune organs, including the kidney marrow and the spleen [22, 23]. The advent of powerful “omics” technologies, including proteomics, has recently demonstrated that immune organs (e.g., the kidney marrow) from young (two-months old) individuals have a proteomic profile compatible with active DNA damage repair and cellular proliferation. On the other hand, immune organs from old (four months old) fish, are characterized by typical inflammation-related terms, such as interleukin-1β synthesis. Plasma proteomics further indicates that plasma from young-adult killifish is characterized by anti-inflammatory terms, such as Annexin 1A, a known NF-kB pathway inhibitor [22]. Conversely, in aged killifish, the plasma reveals marked increases in acute-phase proteins closely associated with inflammatory processes, including elevated levels of clotting factors like FGG and FGB, as well as heightened presence of components from the complement system such as C8G and C8B. These findings collectively underscore the organism's heightened response to wounds and infections. To note, blood from old killifish, compared to blood from young-adult individuals, presents hyperinsulinemia – which is strongly associated with human aging [24] – and higher levels of IGF1.

Both systemic (serum) and organ-specific proteomics in killifish show spontaneous onset of molecular hallmarks of aging associated with inflammation, reduced DNA repair, response to pathogens and metabolic dysfunctions. At the cellular level, cytometric analysis in the kidney marrow of aging killifish indicates that markers of DNA double-strand breaks (γH2AX) and cellular senescence (SA- β-Gal) accumulate in cells from aged killifish, and in particular in progenitor cells [22].

Overall, killifish appear to recapitulate several markers associated with aging during mammalian hematopoiesis, including genotoxic and replicative stress [25].

### The killifish IgH locus architecture and evolution

Lymphocyte-based adaptive immunity is a distinct trait associated with the evolutionary success of jawed vertebrates [26]. The evolution of adaptive immunity allowed jawed vertebrates to recognize and respond to a vast range of pathogens and parasites. Lymphocyte-mediated adaptive immune recognition enabled for the establishment of long-lasting immunological memory, resulting in effective and precise immune responses upon re-exposures to previously encountered immunological threats. The evolution of this formidable line of defense and immune recognition has also been associated with the establishment of highly complex commensal vertebrate-specific microbial communities [27]. Age-dependent dysfunctions in lymphocyte-mediated adaptive immunity has been demonstrated in humans and mice [28, 29], and has been associated with impaired responses to infectious agents – as well as to vaccines – in the elderly population [30]. A key genomic locus that mediates adaptive immune responses is the Immunoglobulin Heavy Chain (IgH) locus. This locus undergoes somatic recombination during B cell maturation and is responsible for the generation of a vast diversity of antibodies in naïve B cells.

**Fig. 1:**
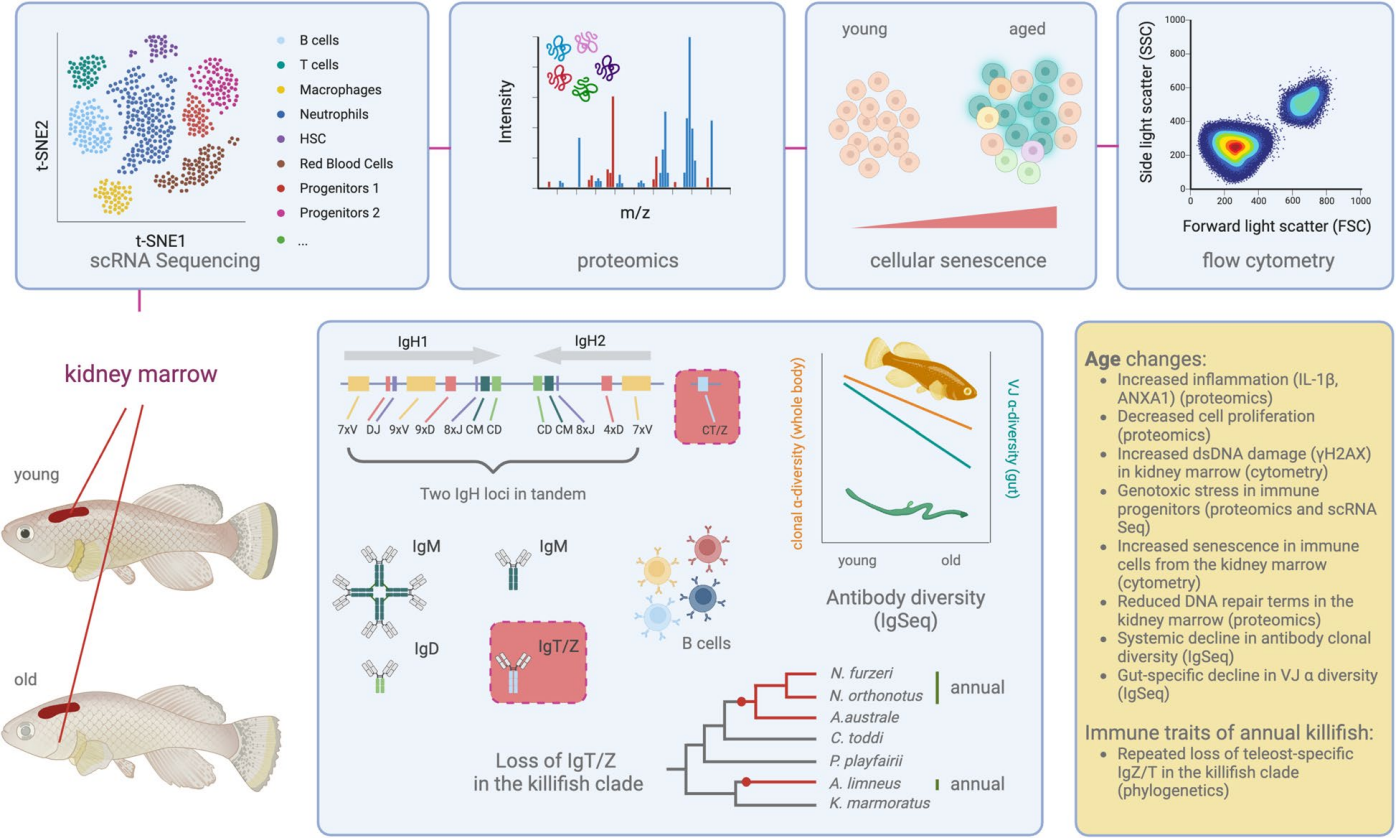
Multi-dimensional molecular profiling of immune aging in turquoise killifish

As vertebrates, killifish are equipped with an IgH locus and cellular machinery able to build a humoral immune response against potentially pathogenic antigens. Bradshaw and Valenzano studied the IgH locus architecture in killifish and showed that indeed killifish have two IgH loci in tandem on chromosome 6, able to generate both IgM (secreted and transmembrane) and IgD antibody isotypes [31] (Fig. 1). To note, killifish of the genus *Nothobranchius*, together with the genus *Aphyosemion* (a genus of non-annual African killifish) and *Austrofundulus* (a genus of annual south American killifish), appear to have completely lost a teleost-specific mucosal antibody isotype, i.e., IgT/Z [31] (Fig. 1). The causes underlying the loss of same antibody isotype in several killifish species remains unclear. Consistent with other teleosts, which do not undergo class-switch recombination [32], also killifish do not show IgG/A/Es.

The genomic structure of the IgH locus among killifishes is highly heterogeneous. Different killifish species present large variation in the IgH locus, which underwent extensive remodeling and diversification throughout the evolution of this clade.

Nonetheless, the assessment of the IgH locus's products, namely antibodies, cannot rely solely on the examination of the genomic locus; it necessitates the complementary analysis of B cell-specific transcripts.

### Aging changes in the killifish immunoglobulin repertoire

During aging, B cells become less effective in establishing powerful humoral immune responses, contributing to higher titers of autoreactive antibodies, increasing the risk for infections and leading to worse vaccine outcomes. Furthermore, aging B cells undergo clonal expansion even without antigenic challenges, leading to lowered primary B cell diversity [33], which might contribute to defective humoral immunity. Human elderly appear to have more specialized and less plastic B cell responses [29]. Like humans, mice also display age-dependent decline in spleen-specific antibody diversity [28]. What about non-mammalian vertebrates? Do teleosts undergo aging in the B cell compartment? Assembling the genomic sequence of the IgH locus’ constant region in turquoise killifish has been instrumental for characterizing the expressed antibody repertoire. By selective amplification and sequencing of B cell-specific transcripts from the IgH locus via IgSeq [34], it was recently possible to study the expressed killifish immunoglobulin diversity [35]. Expression data via IgSeq, enabled to estimate the entropy of turquoise killifish’s productive IgH repertoire, which amounts to 23 bits [35]. Each adult killifish is therefore able to generate ~ 10^7^ possible unique sequences before selection in the primary lymphoid organ. While 23 bits represents a rather diverse range of possible primary antibodies, the killifish IgH locus has a generative entropy that is 50 times smaller than the human’s [36]. Bradshaw et al. discovered that the entropy of the antibody generative process does not decline during killifish aging, suggesting that killifish might retain the capacity to generate novel naïve B cells throughout life [35]. However, while the primary killifish antibody repertoire appears to be unaffected during aging, the diversity of the largest B cell clones rapidly declines during aging. Furthermore, the sequence diversity (VJ α-diversity) of intestinal B cell antibodies does undergo a dramatic age-specific decline. One possible explanation could be that the constant antigen exposure of the intestinal mucosa might lead to extensive clonal expansion, resulting in a lower relative number of unique VJ combinations in older killifish. Another plausible reason for the age-dependent decline in gut VJ α-diversity could be that the intestinal epithelium has a smaller clone-size distribution than the whole-body repertoire, hence sampling the repertoire diversity towards the larger-size clones [35].

As repertoire diversity declines during aging in killifish, the pairwise inter-individual repertoire diversity (repertoire dissimilarity index, or RDI), increases in aged fish. Hence, young killifish have more similar antibody repertoires, while older killifish have more individualized antibody repertoires, which might reflect the individual-specific exposure to antigens occurring throughout life [35].

To address which immune-system-wide functional terms correlated with high antibody repertoire diversity (from Shannon entropy to diversity measures accounting for relative abundance), Bradshaw et al. conducted a differential expression analysis of total intestinal RNA transcripts, with respect with antibody repertoire diversity, factoring out age [35]. This analysis showed that B cell functional terms, such as “B cell signaling pathway”, and “B cell proliferation”, alongside “microglia cell activation” (possibly enteric glia) and “erythrocyte development” ranked among the top GO terms.

Overall, killifish undergo dramatic antibody diversity loss during aging, and antibody diversity per se strongly correlates with immune terms associated with B cell activation and proliferation. Strikingly, several features of aging in the killifish B cell compartment resemble mechanistic changes identified in human aging, including narrowing of clonotypic diversity [37].

### Microbiome age changes and immune cell function

Smith et al., found that young-adult vs. old turquoise killifish have significant differences in their gut microbiome, including declined taxonomic richness, increased prevalence of pathogenic Proteobacteria, and increased pairwise microbial taxonomic diversity [38]. Aging-dependent shifts in microbiome composition have also been reported in Drosophila [39]. Similarly, mice undergo taxonomic and functional changes in the microbiome during aging [40]. In humans, aging has been associated with increased microbiome uniqueness [41, 42]. The age-dependent changes in microbial diversity observed in killifish occur alongside intestinal transcriptional changes associated with increased inflammation, activation of innate immune responses, as well as reduced cell adhesion. In a heterochronic transplantation experiment where gut content from young fish was transplanted into aged killifish, a striking increase in lifespan was observed when compared to mid-age control fish that received gut content from age-matched peers. This effect was accompanied by distinctive transcriptional signatures indicating reduced intestinal inflammation, increased B cell activation (notably POU2AF1), as well as elevated levels of bactericidal lipid-binding serum glycoproteins, such as BPI, and antimicrobial peptides, for instance, HAMP [38]. Together, heterochronic microbiome transfers appears to induce intestinal transcriptional changes associated with lowered inflammation and improved immune protection from pathogens. Recent work in mice suggests that heterochronic stool transplantation in mice ameliorates immune function and brain phenotypes [43, 44]. Furthermore, targeted restoration of the healthy microbiome in humans has been suggested as a promising strategy to promote healthy aging [45].

### Cancer in killifish

Annual killifish of the genus *Nothobranchius* have long been reported to carry a remarkably high incidence of spontaneous neoplastic transformations [9–11], which have been associated with thymic involution and malignant changes, including lymphomas. The most affected organs appear to be the liver and the kidney marrow, i.e., the main teleost hematopoietic organ [46]. However, a recent study issues a caveat regarding the reliance on conventional histological methods for the diagnosis of tissue lesions as neoplastic. It underscores that host defense responses to mycobacterial infections can exhibit neoplastic-like characteristics, such as the activation of mononuclear phagocytic processes and the formation of inflammatory granulomas [47]. While responses to mycobacterial infections might be mistaken as neoplasias, their increased occurrence in elderly fish are nonetheless suggestive of age-dependent immunological dysfunctions.

The common platyfish (*Xiphophorys maculatus*) and the green swordtail (*Xiphophorus helleri*), which belong to the same order (Cyprinodontiformes) of annual killifish of the genus *Nothobranchius*, have been extensively studied as models of genetically and environmentally-induced melanoma [48, 49]. Zebrafish and medaka, on the other hand, which are powerful models for experimentally-induced genetic manipulations, have been developed as models for oncogene-induced melanoma (e.g., RAS-RAF) [48, 50]. Whether killifish naturally manifest unequivocal cancerous lesions requires further comprehensive molecular validation. Nevertheless, killifish can become a promising cancer model due to their intrinsic characteristics. Like platyfish, they exhibit spontaneous pigment aberrations [15], while sharing with medaka and zebrafish the amenability to genetic manipulation through transgenesis and genome editing [51–54]. Furthermore, with a short natural lifespan and an organ-wide emergence of aging-related changes, turquoise killifish offer the opportunity to study the impact of aging on organ-specific tumorigenesis.

Future research will be needed to shed light on the onset of spontaneous malignant transformations in aging killifish.

### Opportunities for interventions

The spontaneous onset molecular, cellular, tissue-specific and systemic changes associated with aging of the immune system, make annual killifish of the genus *Nothobranchius* an ideal experimental model for immune-targeted interventions aimed at ameliorating health through the aging process.

A recent preprint by Morabito et al., showed that while primary immune cells extracted from the killifish kidney marrow from young vs. old turquoise killifish respond differently to LPS (endotoxin) induction, the application of the senolytic drug fisetin to cells from old killifish donors leads to cellular responses to LPS comparable to those of young cells [22]. Senescent cells appear to dramatically accumulate in aging killifish across organs [55, 56]. The application of senolytics, such as a cocktail of quercetin and dasatinib, led to enhanced neurogenesis after traumatic brain injury [57]. However, whether senolytics lead to improved immune-aging phenotypes also in vivo, is still unclear.

## Conclusions

In summary, annual killifish undergo substantial transformations in immune aging phenotypes. These transformations encompass shifts in cellular homeostasis within their primary hematopoietic organ and profound alterations in immune effector cells, including lymphocytes within mucosal organs. Additionally, they exhibit systemic changes characterized by heightened inflammatory processes.

The unique combination of their short natural lifespan, a broad range of spontaneous aging-related immune transformations, and their experimental accessibility, makes annual killifish of the genus *Nothobranchius* exceptional candidates for discovering basic molecular, cellular and systemic changes underlying vertebrate immune aging.

Future and ongoing research endeavors will be essential to shed light on multiple aspects related with immune aging in killifish. A comparative analysis of adult hematopoiesis and immune homeostasis among short-lived and long-lived killifish, as well as between killifish and other teleosts, will be necessary to understand whether killifish hematopoiesis has unique features that lead to the rapid onset of age-related immune and systemic dysfunctions. We still ignore whether sustained innate immune system activation impacts endothelial, adipose cells, as well as fibroblasts [58]. We don’t know whether upon immune stimulation, older killifish undergo exaggerated innate responses, such as “cytokine storms” and sepsis, even in response to vaccination [59–61]. It is yet to be determined if aging killifish accumulate age-associated B cells (ABCs) [62, 63], undergo autoimmunity – perhaps associated with thymic involution – and if aging affects their T cell compartment.

The deep evolutionary conservation of basic vertebrate immune aging mechanisms shared between mammals and killifish positions these naturally short-lived vertebrates as a powerful platform for testing interventions that may prove effective across vertebrates, including humans. Killifish can be ideal experimental animals to test the impact of immune therapy in the context of aging and aging-related disease models. Building upon established interventions, killifish offer a valuable platform for evaluating the effects of dietary modifications, medications, microbiome manipulations, precise genome editing through CRISPR/Cas9 technology [51, 53], vaccines, and stem cell transplantations [64], in alleviating immune system aging and its associated health implications.

## References

[1] Huxley AL: After many a summer: Chtto & Windus; 1939.

[2] Woodhead AD (1998). Aging, the fishy side: an appreciation of Alex Comfort's studies. Exp Gerontol.

[3] Walford RL, Liu RK, Troup GM, Hsiu J (1969). Alterations in soluble/insoluble collagen ratios in the annual fish, Cynolebias bellottii, in relation to age and environmental temperature. Exp Gerontol.

[4] Bidder GP (1932). Senescence. Br Med J.

[5] Liu RK, Walford RL (1970). Observations on the lifespans of several species of annual fishes and of the world's smallest fishes. Exp Gerontol.

[6] Liu RK, Leung BE, Walford RL (1975). Effect of temperature-transfer on growth of laboratory populations of a South American annual fish Cynolebias bellottii. Growth.

[7] Liu RK, Walford RL (1975). Mid-life temperature-transfer effects on life-span of annual fish. J Gerontol.

[8] Robertson, Robertson OH: Aging in cold blooded animals: MSS Information Corp; 1974.

[9] Markofsky J, Milstoc M (1979). Histopathological observations of the kidney during aging of the male annual fish Nothobranchius guentheri. Exp Gerontol.

[10] Markofsky J, Milstoc M (1979). Aging changes in the liver of the male annual cyprinodont fish. Nothobranchius guentheri Exp Gerontol.

[11] Cooper EL, Zapata A, Garcia Barrutia M, Ramirez JA (1983). Aging changes in lymphopoietic and myelopoietic organs of the annual cyprinodont fish. Nothobranchius guentheri Exp Gerontol.

[12] Cooper EL, Walford RL (1982). New perspectives on aging and immunity: lower animals, ontogeny, phylogeny and immunoendocrinology. Dev Comp Immunol.

[13] Walford RL (1964). The Immunologic Theory of Aging. Gerontologist.

[14] Effros RB (2005). Roy Walford and the immunologic theory of aging. Immun Ageing.

[15] Cellerino A, Valenzano DR, Reichard M (2016). From the bush to the bench: the annual Nothobranchius fishes as a new model system in biology. Biol Rev Camb Philos Soc.

[16] Furness AI (2016). The evolution of an annual life cycle in killifish: adaptation to ephemeral aquatic environments through embryonic diapause. Biol Rev Camb Philos Soc.

[17] Vrtilek M, Zak J, Psenicka M, Reichard M (2018). Extremely rapid maturation of a wild African annual fish. Curr Biol.

[18] Nezhybová V, Reichard M, Blažek R R, Ondračková M (2017). Metazoan parasites of African annual killifish (Nothobranchiidae): abundance, diversity, and their environmental correlates. biotropica.

[19] Cui R, Medeiros T, Willemsen D, Iasi LNM, Collier GE, Reichard M,  Valenzano DR (2019). Relaxed Selection Limits Lifespan by Increasing Mutation Load. Cell.

[20] Willemsen D, Cui R, Reichard M, Valenzano DR. Intra-species differences in population size shape life history and genome evolution. Elife. 2020;9. 10.7554/eLife.55794 PMC7462614 32869739

[21] Cui R, Tyers AM, Malubhoy ZJ, Wisotsky S, Valdesalici S, Henriette E, Kosakovsky Pond SL, Valenzano DR (2021). Ancestral transoceanic colonization and recent population reduction in a nonannual killifish from the Seychelles archipelago. Mol Ecol.

[22] Morabito G, Dönertas HM, Sperti L, Seidel J, Poursadegh A, Poeschla M, Valenzano DR: Spontaneous onset of cellular markers of inflammation and genome instability during aging in the immune niche of the naturally short-lived turquoise killifish (*Nothobranchius furzeri*). bioRxiv 2023:2023.2002.2006.527346.

[23] Xu A, Teefy BB, Lu RJ, Nozownik S, Tyers AM, Valenzano DR, Benayoun BA: Transcriptional profiling of aging tissues from female and male African turquoise killifish. bioRxiv 2023:2023.2006.2020.545766. 10.1038/s41597-023-02609-x PMC10570339 37828039

[24] Gumbiner B, Polonsky KS, Beltz WF, Wallace P, Brechtel G, Fink RI (1989). Effects of aging on insulin secretion. Diabetes.

[25] Flach J, Bakker ST, Mohrin M, Conroy PC, Pietras EM, Reynaud D, Alvarez S, Diolaiti ME, Ugarte F, Forsberg EC (2014). Replication stress is a potent driver of functional decline in ageing haematopoietic stem cells. Nature.

[26] Bagic M, Valenzano DR: Population size shapes the evolution of lifespan. bioRxiv 2022:2022.2012.2017.520867.

[27] Popkes M, Valenzano DR (1808). Microbiota-host interactions shape ageing dynamics. Philos Trans R Soc Lond B Biol Sci.

[28] Monzo C, Gkioni L, Beyer A, Valenzano DR, Gronke S, Partridge L (2023). Dietary restriction mitigates the age-associated decline in mouse B cell receptor repertoire diversity. Cell Rep.

[29] de Bourcy CF, Angel CJ, Vollmers C, Dekker CL, Davis MM, Quake SR (2017). Phylogenetic analysis of the human antibody repertoire reveals quantitative signatures of immune senescence and aging. Proc Natl Acad Sci U S A.

[30] Lee JL, Linterman MA (2022). Mechanisms underpinning poor antibody responses to vaccines in ageing. Immunol Lett.

[31] Bradshaw WJ, Valenzano DR (1927). Extreme genomic volatility characterizes the evolution of the immunoglobulin heavy chain locus in cyprinodontiform fishes. Proc Biol Sci.

[32] Wakae K, Magor BG, Saunders H, Nagaoka H, Kawamura A, Kinoshita K, Honjo T, Muramatsu M (2006). Evolution of class switch recombination function in fish activation-induced cytidine deaminase. AID Int Immunol.

[33] Martin V, Bryan Wu YC, Kipling D, Dunn-Walters D (2015). Ageing of the B-cell repertoire. Philos Trans R Soc Lond B Biol Sci.

[34] Weinstein JA, Jiang N, White RA, Fisher DS, Quake SR (2009). High-throughput sequencing of the zebrafish antibody repertoire. Science.

[35] Bradshaw WJ, Poeschla M, Placzek A, Kean S, Valenzano DR. Extensive age-dependent loss of antibody diversity in naturally short-lived turquoise killifish. Elife. 2022;11. 10.7554/eLife.65117 PMC8880994 35129436

[36] Elhanati Y, Sethna Z, Marcou Q, Callan CG, Mora T, Walczak AM (2015). Inferring processes underlying B-cell repertoire diversity. Philos Trans R Soc Lond B Biol Sci.

[37] Cancro MP, Hao Y, Scholz JL, Riley RL, Frasca D, Dunn-Walters DK, Blomberg BB (2009). B cells and aging: molecules and mechanisms. Trends Immunol.

[38] Smith P, Willemsen D, Popkes M, Metge F, Gandiwa E, Reichard M, Valenzano DR. Regulation of life span by the gut microbiota in the short-lived African turquoise killifish. Elife. 2017;6. 10.7554/eLife.27014 PMC5566455 28826469

[39] Clark RI, Salazar A, Yamada R, Fitz-Gibbon S, Morselli M, Alcaraz J, Rana A, Rera M, Pellegrini M, Ja WW (2015). Distinct Shifts in Microbiota Composition during Drosophila Aging Impair Intestinal Function and Drive Mortality. Cell Rep.

[40] Langille MG, Meehan CJ, Koenig JE, Dhanani AS, Rose RA, Howlett SE, Beiko RG (2014). Microbial shifts in the aging mouse gut. Microbiome.

[41] Wilmanski T, Diener C, Rappaport N, Patwardhan S, Wiedrick J, Lapidus J, Earls JC, Zimmer A, Glusman G, Robinson M (2021). Gut microbiome pattern reflects healthy ageing and predicts survival in humans. Nat Metab.

[42] O'Toole PW, Jeffery IB (2015). Gut microbiota and aging. Science.

[43] Boehme M, Guzzetta KE, Bastiaanssen TFS, van de Wouw M, Moloney GM, Gual-Grau A, Spichak S, Olavarria-Ramirez L, Fitzgerald P, Morillas E (2021). Microbiota from young mice counteracts selective age-associated behavioral deficits. Nat Aging.

[44] Stebegg M, Silva-Cayetano A, Innocentin S, Jenkins TP, Cantacessi C, Gilbert C, Linterman MA (2019). Heterochronic faecal transplantation boosts gut germinal centres in aged mice. Nat Commun.

[45] Ghosh TS, Shanahan F, O'Toole PW (2022). The gut microbiome as a modulator of healthy ageing. Nat Rev Gastroenterol Hepatol.

[46] Di Cicco E, Tozzini ET, Rossi G, Cellerino A (2011). The short-lived annual fish Nothobranchius furzeri shows a typical teleost aging process reinforced by high incidence of age-dependent neoplasias. Exp Gerontol.

[47] Dykova I, Zak J, Reichard M, Souckova K, Slaby O, Bystry V, Blazek R (2021). Histopathology of laboratory-reared Nothobranchius fishes: Mycobacterial infections versus neoplastic lesions. J Fish Dis.

[48] Patton EE, Mitchell DL, Nairn RS (2010). Genetic and environmental melanoma models in fish. Pigment Cell Melanoma Res.

[49] Powell DL, Garcia-Olazabal M, Keegan M, Reilly P, Du K, Diaz-Loyo AP, Banerjee S, Blakkan D, Reich D, Andolfatto P (2020). Natural hybridization reveals incompatible alleles that cause melanoma in swordtail fish. Science.

[50] White R, Rose K, Zon L (2013). Zebrafish cancer: the state of the art and the path forward. Nat Rev Cancer.

[51] Harel I, Valenzano DR, Brunet A (2016). Efficient genome engineering approaches for the short-lived African turquoise killifish. Nat Protoc.

[52] Valenzano DR, Sharp S, Brunet A (2011). Transposon-Mediated Transgenesis in the Short-Lived African Killifish Nothobranchius furzeri, a Vertebrate Model for Aging. G3 (Bethesda).

[53] Rozenberg I, Moses E, Harel I (2023). CRISPR-Cas9 Genome Editing in Nothobranchius furzeri for Gene Knockout and Knock-In. Cold Spring Harb Protoc.

[54] Krug J, Perner B, Albertz C, Morl H, Hopfenmuller VL, Englert C. Generation of a transparent killifish line through multiplex CRISPR/Cas9 mediated gene inactivation. Elife. 2023;12. 10.7554/eLife.81549 PMC10010688 36820520

[55] Ahuja G, Bartsch D, Yao W, Geissen S, Frank S, Aguirre A, Russ N, Messling JE, Dodzian J, Lagerborg KA et al. Loss of genomic integrity induced by lysosphingolipid imbalance drives ageing in the heart. EMBO Rep. 2019;20(4). 10.15252/embr.201847407 PMC6446199 30886000

[56] Valenzano DR, Terzibasi E, Cattaneo A, Domenici L, Cellerino A (2006). Temperature affects longevity and age-related locomotor and cognitive decay in the short-lived fish Nothobranchius furzeri. Aging Cell.

[57] Van Houcke J, Marien V, Zandecki C, Ayana R, Pepermans E, Boonen K, Seuntjens E, Baggerman G, Arckens L (2023). A short dasatinib and quercetin treatment is sufficient to reinstate potent adult neuroregenesis in the aged killifish. NPJ Regen Med.

[58] Nidadavolu LS, Walston JD (2021). Underlying Vulnerabilities to the Cytokine Storm and Adverse COVID-19 Outcomes in the Aging Immune System. J Gerontol A Biol Sci Med Sci.

[59] Cunha LL, Perazzio SF, Azzi J, Cravedi P, Riella LV (2020). Remodeling of the Immune Response With Aging: Immunosenescence and Its Potential Impact on COVID-19 Immune Response. Front Immunol.

[60] Kasler H, Verdin E (2021). How inflammaging diminishes adaptive immunity. Nat Aging.

[61] Vitalle J, Perez-Gomez A, Ostos FJ, Gasca-Capote C, Jimenez-Leon MR, Bachiller S, Rivas-Jeremias I, Silva-Sanchez MDM, Ruiz-Mateos AM, Martin-Sanchez MA et al: Immune defects associated with lower SARS-CoV-2 BNT162b2 mRNA vaccine response in aged people. JCI Insight. 2022;7(17). 10.1172/jci.insight.161045 PMC9536264 35943812

[62] Frasca D, Romero M, Garcia D, Diaz A, Blomberg BB (2021). Hyper-metabolic B cells in the spleens of old mice make antibodies with autoimmune specificities. Immun Ageing.

[63] Ma S, Wang C, Mao X, Hao Y (2019). B Cell Dysfunction Associated With Aging and Autoimmune Diseases. Front Immunol.

[64] Rozenberg I, Atlan T, Franek R, Moses E, Oron-Gottesman A, Chrzanowski H, Harel I: Exploring life-long tissue homeostasis through lineage tracing and cell transplantation. bioRxiv 2023:2023.2005.2001.538839.

